# Evaluation of Microhardness of Two Bulk-fill Composite Resins Compared to a Conventional Composite Resin on surface and in Different Depths

**DOI:** 10.30476/DENTJODS.2021.87669.1278

**Published:** 2022-03

**Authors:** Keyvan Saati, Sheida Khansari, Farnaz Mahdisiar, Sara Valizadeh

**Affiliations:** 1 Dept. of Restorative Dentistry, Dental Branch, Islamic Azad University of Medical Sciences, Tehran, Iran; 2 Dental Branch, Islamic Azad University of Medical Sciences, Tehran, Iran; 3 Dental Research Center, Dentistry Research Institute, Dept. of Restorative Dentistry, School of Dentistry, Tehran University of Medical Sciences, Tehran, Iran

**Keywords:** Bulk-fill, Composite resins, Curing depth, Microhardness, Polymerization

## Abstract

**Statement of the Problem:**

One of the problems with light-cured composite resins is the limitation and inadequate depth of curing and polymerization, resulting in low surface microhardness and restoration failure.

**Purpose:**

The present study aimed to compare the surface microhardness of two different bulk-fill composite resins and one conventional composite resin using the Vickers microhardness test.

**Materials and Method:**

In the present *in vitro* study, 108 samples from two different bulk-fill composite resins (Tetric N Ceram and Xtrafil) and one conventional composite resin (Filtek Z250) were prepared in metallic molds (2×4×10 mm) (n=36 for each composite resin). Six samples from each composite resin (n=6) underwent a hardness measurement test at specific depths (0.1, 1, 2, 3, 4 and 5mm). The samples were then stored at 37ºC for 24 hours, followed by a microhardness test at the depths mentioned above.

**Results:**

In all the composite resin samples, microhardness decreased with an increase in depth. The highest microhardness was recorded in Filtek Z250, followed by Xtrafil, with no significant difference. The lowest microhardness was recorded in Tetric N Ceram bulk-fill. Both bulk-fill composite resins at all the depths exhibited depth-to-surface standard microhardness (>80%).

**Conclusion:**

According to the results, both evaluated bulk-fill composite resins exhibited favorable surface microhardness up to a depth of 5 mm.

## Introduction

Composite resins are widely used in dentistry. One of the concerns with the use of light-cured composite resins is their limited light penetration, resulting in inadequate curing depth and polymerization [ 1 ].

Adequate polymerization is a vital factor in achieving favorable mechanical properties, which ensures satisfactory clinical efficacy of composite resin restorations [ 2 ].

The release of uncured monomers, followed by decreased biocompatibility of restorations and compromised physical properties, including low color stability, result from inadequate polymerization of composite resin restorations [ 3 ].

Various factors affect the photo-polymerization of composite resins, including composite resin type, its color and translucency, the thickness of each composite resin layer, the distance between the tip of the light-conducting nozzle and the composite resin surface, the type of the light-curing unit, curing parameters, irradiation mode, photo initiators, the size and distribution of fillers, and viscosity [ 4 - 5 ].

Composite resins can be cured at specific depths, depending on the penetration of light into the composite resin bulk. The light energy of light-curing unit decreases gradually as it transverses through the composite resin bulk [ 6 ]. Several techniques have been proposed to overcome this problem, including the layering technique for composite resin placement or the adjustment of the irradiation mechanisms. However, the layering technique is time-consuming, with a high risk of air bubble entrapment and contamination [ 7 ].

Bulk-fill composite resins were introduced to overcome the problems above. According to the manufacturer, these composite resins, which have become very popular with dentists due to their ease of use, have a 4-mm curing depth. Besides, this bulk of composite resin can be cured in one stage because it has low polymerization shrinkage and minimal polymerization stress [ 8 ].

In bulk-fill composite resins, high translucency, high monomer technology, modifications in fillers and use of new photo initiators have decreased polymerization stresses and increased the curing depth, with a significant adaptation with the cavity walls as an advantage. However, these composite resins have some disadvantages, including postoperative sensitivity, microleakage and debonding [ 9 - 10 ].

Surface hardness is used to predict materials’ wear resistance to abrasion and abrasive caused by opposing teeth. The depth-to-surface microhardness ratio of composite resins is 0.8–0.85, so that it can be ensured that the base has adequately been polymerized [ 11 ]. 

Microhardness is defined as the resistance against penetration or permanent indentation of the surface, which is a criterion for resistance against plastic deformation and is calculated by dividing the force by the indented surface area. Vickers test is one of the most common tests in this respect [ 11 - 12 ]. The evaluation of the curing depth of composite resins by measuring the hardness is imperative since there is a relationship between an increase in hardness and curing depth [ 12 ].

Although the clinical use of bulk-fill composite resins is increasing, several previous studies have not fully confirmed their mechanical properties [ 8 , 13 ]. Therefore, it is necessary to evaluate these properties in composite resins, including degree of conversion (DC) or surface hardness, especially in the long term [ 10 , 14 ].

Since there is a lack of adequate data in this filed, and considering the discrepancies about adequate curing depth and durability of bulk-fill composite resins in the use of these composite resins with >2 mm depths, the present study aimed to compare the microhardness of two different types of bulk-fill composite resins and one conventional composite resin at different depths by using Vickers microhardness test. The null hypothesis was that all composites have the same Vickers microhardness in different evaluated depths.

## Materials and Method

In the present *in vitro* study, samples of Tetric N Ceram bulk-fill and Xtrafil bulk-fill and Filtek Z250 conventional composite resins were fabricated using bronze molds. Table 1 presents the characteristics of the composite resins evaluated.

**Table 1:** The characteristics of conventional and bulk-fill composite resins under study

Commercial Brand	Type of Composite	Manufacturer	Composition	Filler Percentage
Tetric N Ceram Bulk Fill	Hybrid	Ivoclar Vivadent AG, Schaan, Liechtenstein	Barium glass, Prepolymer, Ytterbium trifluoride, Mixed oxide Bis-GMA, DMA	75–77 wt%
				53–55 vol%
Xtrafil	Hybrid	VOCO Cuxhaven, Germany	Barium-boron- aluminosilicate glass, Bis-GMA, UDMA, TEGDMA	86 wt.%
				70.1 vol%
Filtek Z250	Micro hybrid	3M ESPE, St. Paul, MN, USA	Zirconia/silica without silane treatment, Bis-GMA, UDMA, Bis-EMA	82 wt.%
				60 vol%

### Sample preparation

A bronze mold, measuring 10 mm in length, 4 mm in width, and 2 mm in depth, was used to produce composite resin samples. The base of the mold had an open-ended cavity to place the restorative material. The base of the mold had been designed to make it possible to adapt it to and hold it on the table of the microhardness machine. On each side of the cavity, a small retentive groove was placed to ensure the stability and no movement of the samples during force application in the test. Besides, the open end of the cavity was placed anterior to the front rim of the table so that it was possible to see the forward and backward movement of the machine to the position zero. The mold was cleaned with cotton soaked with alcohol between the different rounds of composite resin placement. The composite resins were placed in the mold. The mold was placed on a glass slab and the composite was packed within it. A translucent celluloid tape and a glass slab were used on the open end of the mold and a 5 Kg weight was used to apply pressure from the top for 3 minutes. This pressure would extrude the extra composite resin, and subsequently, a smooth surface would be achieved; moreover, it would ensure uniform thickness of sample and eliminate the voids. The mold was closed from each side with butterfly screws, and the composite resin was light-cured from the exposure side for 20 seconds. 

An LED light-curing unit (BluePhase N, Ivoclar Vivadent AG, Schaan, Liechtenstein) was used in the present study, delivering light with a wavelength of 385–515 nm at 1200 mW/cm^3^ intensity. The light-curing unit was checked with a radiometer (Model 100; Optilux, SDS, Kerr, Orange, CA, USA) after five curing rounds. The tip of the light-conducting nozzle was placed at contact with the glass slab to standardize the distance between the nozzle and the composite resin surface during polymerization. Thirty-six samples were fabricated in this method. After retrieving the samples from the molds, the lateral aspect of the samples was marked to identify the upper and lower sides of the samples. Then the upper and lower surfaces of the samples were polished with 800-, 1000- and 1200-grit polishing paper (Struers, Ballerup, Denmark) to achieve a smooth surface. The sample dimensions were measured with a digital caliper (Mitutoyo, Tokyo, Japan) with 0.01 mm accuracy after polishing. Then, the samples were stored in water at 37ºC for 24 hours.

### Surface Microhardness measurement

A Vickers microhardness equipment (Bareiss, Germany) was used to determine the microhardness of samples at 0.1, 1, 2, 3, 4 and 5mm depths (comprising of six samples for measuring microhardness at each depth). A fixed force of 300 gr was applied for 15 seconds in each indentation to determine the samples’ microhardness.

Measurements began from 0.1 mm from the light-cured surface to avoid the air-inhibited layer and ended at a depth of 5 mm. Three indentations were produced at each layer at a distance of 0.2mm. The mean of these three values was calculated and reported as the hardness value. The measurements were carried out at 40× for all the samples. 

### Statistical Analysis

The data were analyzed with SPSS 25.0. Since data were distributed normally, Two-way ANOVA was used to compare microhardness changes between the groups. In addition, post hoc Tukey tests were used for two-by-two comparisons of groups. The acceptable type I error was set at 0.05 in this study (α=0.05).

## Results

Table 2 presents microhardness values in terms of the composite resin type and the evaluated depth. 

**Table 2:** The microhardness of composite resins evaluated in the present study at different depths

Composite resin type	Composite Thickness (mm)	Vickers Microhardness (MPa)
Filtek Z250	0.1	98.8±3.8 a
	1	93±2 b
	2	91.1±1 c
	3	81±5.7 d
	4	No curing
	5	No curing
Tetric N Ceram Bulk fill	0.1	68.1±10 ^g^
	1	66.6±11.8 ^h^
	2	63.6±10.8 ^i^
	3	64.2±11.4 ^j^
	4	60.8±14.3 ^k^
	5	55.1±13.2 ^l^
Xtrafil	0.1	99±8.3 ^a^
	1	99.1±9.2 ^b^
	2	96.3±9.4 ^c^
	3	92.3±8.5 ^d^
	4	88.5±9.01 ^e^
	5	86.7±14.6 f

* Different superscript letters show significant differences between microhardness of groups.

Two-way ANOVA test showed that composite type (*p*= 0.0001) and depth of sample (*p*= 0.008) had a significant effect on composite microhardness but their interaction was not significantly affected on microhardness. In other words, all the composite resins tested in this study had the same behavior in different depths regarding microhardness. 

The results showed that the hardness decreased with an increase in depth (thickness) in Filtek Z250 composite resin, with hardness values of 98.8 and 81 N/mm^2^ at 0.1- and 3-mm depths, respectively (*p*< 0.01). It was not possible to measure microhardness at 4- and 5-mm depths because composite resins had not been cured. However, at all the similar depths, the depth-to-surface microhardness ratio standard level was >80% according to ISO 4049.

Microhardness values in the Tetric N Ceram bulk-fill composite resin showed that the microhardness decreased with an increase in thickness, with 68.1 and 55.1 N/mm^2^ on the surface and 5mm depth, respectively (*p*< 0.01). 

At all the depths in this composite resin, the depth-to-surface microhardness ratio was at a favorable level (>80%).

In the Xtrafil composite resin, microhardness decreased with an increase in thickness, with 99 and 6.7 N/mm^2^ on the surface and 5-mm depth, respectively. The decrease in microhardness with an increase in thickness was statistically significant (*p*< 0.01). In this composite resin, too, the microhardness was at a favorable level in all the thicknesses.

Two by two comparison of composite types showed no significant difference between Z250 and Xtrafil (*p*= 0.469) microhardness in the same depth, however, two other composites had no significant difference (*p*= 0.0001) in this respect.

## Discussion

The present study aimed to evaluate the effect of different thicknesses on the microhardness of bulk-fill composite resins. In the present study, Filtek Z250 composite resin was used as a reference. Filtek Z250 is a microhybrid and opaque composite resin, designed for anterior and posterior restorations. It contains inorganic silica and zirconia fillers, and particles resulting from the abrasion of glass, quartz and Bis-EMA, Bis-UDMA, and Bis-GMA resin matrix. This product has excellent resistance to abrasion and favorable mechanical properties and is suitable for areas under stress. The present study showed that bulk-fill composite resins are properly polymerized up to a depth of 4 mm. Studies by Alrahalah *et al*. [ 15 ], Alshali *et al*. [ 16 ], and Garounshit *et al*. [ 17 ] are consistent with the present study. They showed that the curing depth of bulk-fill composite resins was adequate, comparable to conventional composite resins, and consistent with the present study.

Different techniques are available to determine the curing depth. Laser and Fourier transform infrared (FTIR) are direct techniques to determine the curing depth, and scraping, microhardness testing, and visual inspection are indirect techniques to determine hardness [ 18 ]. In this study, Vickers Microhardness used for evaluating DC because this test is widely used to examine resin composite polymerization due to the direct correlation between the microhardness of a composite and the DC [ 19 ]. 

In the present study, Xtrafil and Z250 composite reins exhibited the highest microhardness and Tetric N Ceram bulk-fill exhibited the lowest microhardness. Filtek Z250 and Xtrafil composite reins exhibited similar microhardness up to a depth of 3 mm, and both had microhardness higher than Tetric N Ceram bulk-fill composite resin. Besides, microhardness decreased with an increase in thickness in all the samples. 

High microhardness in Xtrafil is consistent with other studies [ 20 - 21 ]. This high microhardness might be attributed to differences in the chemical composition of resin matrix and the viscosity of monomers. Besides, the flexibility of the monomer’s chemical structure might affect the curing depth of composite resin. The resin matrix of Xtrafil is composed of bis-GMA, UDMA, and TEGDMA. TEGDMA is considered a diluting monomer and exhibits the highest DC among composite resin monomers. When bis-GMA is diluted with a low-viscosity monomer, there is a synergistic effect on DC and curing depth. Therefore, a high concentration of TEGDMA might be a reason for a high curing depth of Xtrafil composite resin [ 21 - 22 ]. In addition, the multi-hybrid filler technology has been used in this material, resulting in a decrease in polymerization shrinkage, a 4-mm curing depth, high surface hardness, and high resistance to abrasion in this material [ 23 ]. The high hardness of the Z250 composite rein might be attributed to its fillers composed of quartz and ceramic particles [ 24 ]. 

The significantly low surface hardness of Tetric N Ceram bulk-fill composite rein might be attributed to the initiator/catalyst system, the type of monomer, and the use of barium glass fillers with re-polymerized particles in the structure of this composite resin [ 22 ]. The parameters affecting microhardness include the shape and distribution of fillers, the shape and density of particles, and the type and concentration of the monomer, which are different in different composite resins [ 25 ].

In all the composite reins evaluated, the surface hardness was significantly higher than that of the depth. Hardness decreased with an increase in thickness, indicating that the surface of composite rein depends on the light intensity at a lower rate because it absorbs the necessary radiation energy due to its vicinity to the light-conducting tip [ 26 - 27 ]. In the present study, in all the composite resins evaluated, the sample’s depth-to-surface microhardness ratio was >80% (almost 88%). Al-Mansour *et al*. [ 28 ] reported that the proper curing depth in Tetric N Ceram bulk-fill composite resin is due to Ivocerin in its structure, which is an initiator with a germanium base. According to the manufacturer, it has a higher curing activity than camphorquinone because generates at least two free radicals for polymerization initialization compared to camphorquinone, the most widely used visible-light photo-initiator in RBCs that generates only one radical [ 1 , 29 ]. Besides, it can initiate polymerization without adding amine by creating two radicals, which is more effective than the camphorquinone system with only one radical. Other studies have shown that despite a high filler content in Tetric N Ceram bulk-fill composite, the depth-to-surface microhardness ratio <80%, which is different from the present study [ 22 , 30 ].

There is another new advancement in this composite resin system, referred to as the Aessenico technology, in which the refractive index of unpolymerized monomers has been modified to match that of the fillers to achieve a proper curing depth in thick layers. This has resulted in a very translucent structure through which light traverses easily without any barrier. This technology, in association with Ivocerin initiators, provides a reliable and fast polymerization, even at tooth–composite resin interface [ 31 ].

Jang *et al*. [ 30 ] compared the Vickers microhardness of four different types of composite resins, including two bulk-fill flowables (Surefil SDR flow and Venus Bulk fill) and one bulk-fill nonflowable (Tetric N-Ceram Bulk fill) and a highly-filled flowable (G-Aneial Universal Flo) with two conventional composites (Tetric Flow, Filtek Supreme Ultra) up to a depth of 4.5 mm. Tetric N Ceram bulk-fill contains translucent filler and matrix that help transmit light through this material. However, the results showed that all the composite resins, except for SDR and Venus Bulkfill, exhibited low microhardness, and Tetric N Ceram bulk-fill exhibited a base microhardness lower than the standard level, despite its high filler content, which might be attributed to the difference in the light-curing unit. An LED light-curing unit (Bluephase, Ivoclar, Vivadent) with a wavelength of 700 nm was used in that study [ 32 - 33 ]. Any discrepancy between the unit’s radiation wavelength and the photoinitiator’s sensitivity might give rise to limitations in the creation of free radicals and a disturbance in the polymerization process [ 34 ]. 

The employment of new resins and modified regulators and fillers has increased the curing depth of bulk-fill composite resins. Besides, the amount of light penetrating the composite resin depends on the amount of the light reflected, scattered, and absorbed and all of these factors depend on the composite resin structure. Composite resins with smaller fillers scatter more light [ 35 ].

Evidence indicates that an increase in filler content decreases translucency due to an increase in light reflection at filler–resin interface. Translucency increases with an increase in filler size. Therefore, the size, radiopacity, translucency, and pigments or filler particles affect the passage of light through the material, which in turn affects the curing depth [ 36 ].

Further studies are suggested to evaluate other bulk-fill composite resins, abrasion resistance, and fracture resistance.

This study was *in vitro*, therefore it may be different from the clinical situation and besides that, there are some factors available in the mouth like saliva, enzymes, different food and beverages with different pH and temperature that affect composite microhardness during the time.

## Conclusion

Under the limitations of the present study, it was concluded that microhardness decreased in all the composite resins with an increase in depth. In both bulk-fill composite resins, the depth-to-surface microhardness ratio in all depth was at a standard level (>80%).
